# Pten knockout in mouse preosteoblasts leads to changes in bone turnover and strength

**DOI:** 10.1093/jbmrpl/ziad016

**Published:** 2024-01-04

**Authors:** Judith Lorenz, Sandy Richter, Anna S Kirstein, Florentien Kolbig, Michèle Nebe, Marco Schulze, Wieland Kiess, Ingo Spitzbarth, Nora Klöting, Diana Le Duc, Ulrike Baschant, Antje Garten

**Affiliations:** Pediatric Research Center, Leipzig University, University Hospital for Children and Adolescents, Department for Child and Adolescent Medicine, 04103 Leipzig, Germany; Pediatric Research Center, Leipzig University, University Hospital for Children and Adolescents, Department for Child and Adolescent Medicine, 04103 Leipzig, Germany; Pediatric Research Center, Leipzig University, University Hospital for Children and Adolescents, Department for Child and Adolescent Medicine, 04103 Leipzig, Germany; Novo Nordisk Foundation Center for Basic Metabolic Research, Faculty of Health and Medical Sciences, University of Copenhagen, DK-2200 Copenhagen, Denmark; Pediatric Research Center, Leipzig University, University Hospital for Children and Adolescents, Department for Child and Adolescent Medicine, 04103 Leipzig, Germany; Pediatric Research Center, Leipzig University, University Hospital for Children and Adolescents, Department for Child and Adolescent Medicine, 04103 Leipzig, Germany; Saxon Incubator for Clinical Translation (SIKT), Leipzig University, 04103 Leipzig, Germany; Pediatric Research Center, Leipzig University, University Hospital for Children and Adolescents, Department for Child and Adolescent Medicine, 04103 Leipzig, Germany; Faculty of Veterinary Medicine, Institute of Veterinary Pathology, Leipzig University, 04103 Leipzig, Germany; Helmholtz Institute for Metabolic, Obesity and Vascular Research (HI-MAG) of the Helmholtz Zentrum München, Leipzig University and University Hospital Leipzig, 04103 Leipzig, Germany; Institute of Human Genetics, Leipzig University, 04103 Leipzig, Germany; Department of Medicine III, Technische Universität Dresden, 01309 Dresden, Germany; Pediatric Research Center, Leipzig University, University Hospital for Children and Adolescents, Department for Child and Adolescent Medicine, 04103 Leipzig, Germany

**Keywords:** bone marrow, stem cells, Pten, osterix, osteoprogenitor

## Abstract

Bone development and remodeling are controlled by the phosphoinositide-3-kinase (Pi3k) signaling pathway. We investigated the effects of downregulation of phosphatase and tensin homolog (Pten), a negative regulator of Pi3k signaling, in a mouse model of Pten deficiency in preosteoblasts. We aimed to identify mechanisms that are involved in the regulation of bone turnover and are linked to bone disorders.

Femora, tibiae, and bone marrow stromal cells (BMSCs) isolated from mice with a conditional deletion of Pten (Pten cKO) in *Osterix/Sp7*-expressing osteoprogenitor cells were compared to Cre-negative controls. Bone phenotyping was performed by μCT measurements, bone histomorphometry, quantification of bone turnover markers CTX and procollagen type 1 N propeptide (P1NP), and three-point bending test. Proliferation of BMSCs was measured by counting nuclei and Ki-67–stained cells. In vitro, osteogenic differentiation capacity was determined by ALP staining, as well as by detecting gene expression of osteogenic markers.

BMSCs from Pten cKO mice were functionally different from control BMSCs. Osteogenic markers were increased in BMSCs derived from Pten cKO mice, while Pten protein expression was lower and Akt phosphorylation was increased. We detected a higher trabecular bone volume and an altered cortical bone morphology in Pten cKO bones with a progressive decrease in bone and tissue mineral density. Pten cKO bones displayed fewer osteoclasts and more osteoblasts (*P* = .00095) per trabecular bone surface and a higher trabecular bone formation rate. Biomechanical analysis revealed a significantly higher bone strength (*P* = .00012 for males) and elasticity of Pten cKO femora. On the cellular level, both proliferation and osteogenic differentiation capacity of Pten cKO BMSCs were significantly increased compared to controls.

Our findings suggest that Pten knockout in osteoprogenitor cells increases bone stability and elasticity by increasing trabecular bone mass and leads to increased proliferation and osteogenic differentiation of BMSCs.

## Introduction

Growth factor signaling through the phosphoinositide-3-kinase (Pi3k) signaling pathway plays an important role in controlling bone development and remodeling. Constitutive activation of Pi3k signaling was shown to lead to an imbalance of bone homeostasis and skeletal overgrowth. Phosphatase and Tensin Homolog deleted on Chromosome 10 (Pten) counteracts the action of Pi3k. Consistent with this, several studies found a correlation between Pten deficiency and skeletal defects.^1–3^ The effects of Pten loss in bone progenitor cells on bone homeostasis and stability are, however, not fully elucidated.

Pten is a tumor suppressor gene that encodes a dual lipid and protein phosphatase. Pten mutations occur in a variety of human cancers, such as prostate, breast, or bone cancer.^4^^,^^5^ Pten counteracts the Pi3k/Akt/mechanistic target of rapamycin (mTOR) growth–promoting signaling cascade, a key proto-oncogenic player in cancer development and progression as well as a regulator of various cellular processes, including metabolism, survival, proliferation, apoptosis, growth, and cell migration.^6^ Pi3k phosphorylates phosphatidylinositol-4,5-bisphosphate (PIP2) to generate phosphatidylinositol-3,4,5-trisphosphate (PIP3). The serine–threonine kinase Akt is a centrally important downstream effector of PIP3. Pten dephosphorylates PIP3, thereby directly opposing the activity of Pi3k.^7^

To further define the role of Pten in bone homeostasis and bone strength, we selectively deleted Pten exon 5 encoding its phosphatase domain in murine osteoprogenitor cells. By crossing Pten loxP mice with transgenic mice expressing the Cre recombinase driven by the promotor of the transcription factor Osterix (Osx)/Sp7^8^ osteoblasts, osteocytes, hypertrophic chondrocytes, and bone marrow stromal cells (BMSCs) ^9^^,^^10^ were targeted. Thus, Pten’s function of dephosphorylating PIP3 to PIP2 and attenuating Pi3k signaling was deactivated in cells of the early osteoprogenitor stage and beyond. Using these Pten conditional KO (Pten cKO) mice, we aimed to address the consequences of enhanced Pi3k signaling in osteoprogenitor cells on bone function and strength. Pten inactivation in Osx-expressing cells led to increased proliferation and osteogenic differentiation of BMSCs. Pten cKO mice exhibited several changes in the trabecular and, even more pronounced, in the cortical structure of the bone with a progressive increase in trabecular bone volume and cortical thickness. Moreover, Pten cKO mice showed a higher bone strength and elasticity in three-point bending conditions. Thus, these findings highlight the importance of Pten in bone development and bone architecture and provide in vivo evidence for a critical role of Pten specifically in cortical bone development and bone strength.

## Results

### Pten downregulation–enhanced Pi3k signaling in BMSCs

Mice with conditional Pten knockout in Osx expressing osteoprogenitors were overtly normal, with overall growth similar to Cre-negative controls (data not shown). We first checked successful Pten deletion in BMSCs derived from Pten cKO and control mice. Pten knockout was shown on genomic DNA level using primers that amplified the region around the targeted Pten exon 5. In BMSCs of Cre-negative control mice, a band corresponding to Pten exon 5 and flanking loxp sites (floxed exon 5) was amplified. In Pten cKO mice, we detected both a lack of exon 5 and the floxed region containing exon 5, indicating that, as expected, the knockout did not occur in the entire BMSC population (Supplemental Figure S1A).

We also noticed that mice developed hyperplastic lymph nodes in the axillaries at an age of approximately 20 wk. We examined multiple sections of lymph node tissue, predominantly mature cells and lymphoid cells in the paracortical and follicular areas and within the sinusoids. We found no organotypic architecture of the lymph node but replacement by diffuse infiltration of predominantly mature CD19+ lymphocytes (data not shown). Male and female Pten cKO mice at 17 and 38 wk also showed enlarged spleens, which could point to hematologic disease (Supplemental Figure S2A). Blood analysis revealed a higher number of lymphocytes and lower number of neutrophils in the blood of Pten cKO mice compared to Cre-negative mice (Supplemental Figure S2B).

**Figure 1:**
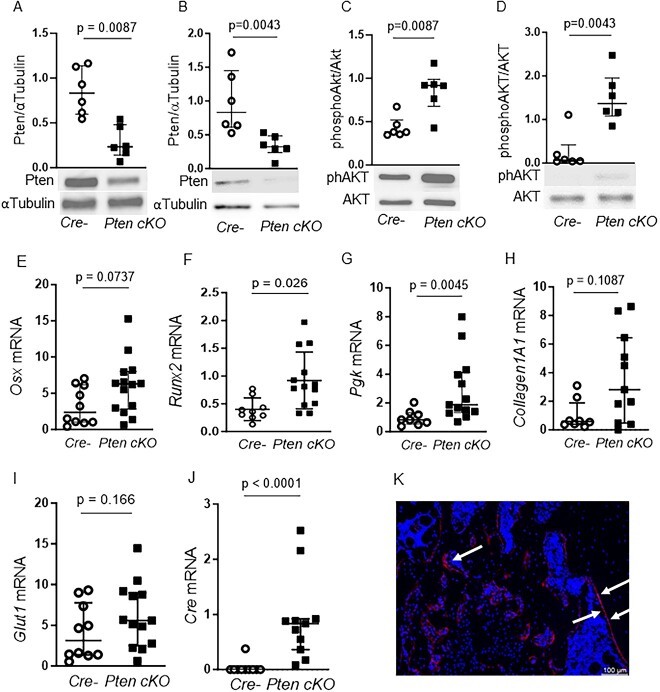
Pten downregulation enhances PI3K signaling in BMSCs*.* Western blot analysis of whole-bone protein (*n* = 4 males, 2 females for Cre neg, *n* = 2 males, 4 females for Pten cKO) and BMSCs (*n* = 3 males, *n* = 3 females per group). Pten protein was significantly reduced in whole-bone lysates (A, *P* = .0087) and in BMSCs (B, *P* = .0043) of Pten cKO mice. α-Tubulin was used as loading control. AKT (Ser473) phosphorylation was approximately doubled in whole-bone lysates (C, *P* = .0087) and in BMSCs (D, *P* = .0043) of Pten cKO mice. Phosphorylated Akt was normalized to total Akt. Data are presented as median and interquartile range, showing all data points. Representative western blot images are shown. Gene expression of (E) Osx, (F) Runx2, (G) Pgk, (H) Col1a1, (I) Glut1, and (J) Cre recombinase in control and Pten cKO BMSCs from male mice (*n* = 8–10 Cre negative and *n* = 11–14 Pten cKO BMSCs). Data are presented as median and interquartile range. (K) A representative image of GFP-positive cells (red) in Pten cKO mTmG mice is shown. Arrows point to GFP-positive areas.

To test whether the restricted Pten downregulation would influence the Pi3k signaling cascade activation, we detected Pten protein levels and subsequent activation of Pi3k downstream target Akt in whole-bone lysates and BMSCs derived from Cre-negative and Pten cKO (*n* = 3 male and *n* = 3 female mice per group) mice. Pten was reduced by 0.5-fold both in whole-bone lysates (Figure 1A, *P* = .0087) and cultured BMSCs (Figure 1B, *P* = .0043) from Pten cKO mice. In line with this, Akt(S473) phosphorylation was approximately doubled in Pten cKO bone lysates (Figure 1C, *P* = .0087) and approximately 1.3-fold higher in BMSCs (Figure 1D, *P* = .0043) from Pten cKO mice.

We next checked if expression of osteogenic marker genes and Pi3k target genes is altered in Pten cKO BMSCs (Figure 1E–J). The expression of the osteogenic markers *Osx* and Runt-related transcription factor (*Runx)2* was higher in Pten-deficient BMSCs compared to control BMSCs (Figure 1E and F). Expression of *Phosphoglycerate kinase (Pgk)*, a target enzyme of Pi3k/Akt signaling, was increased (Figure 1G) and *Collagen(Col)1a1* showed a trend toward higher expression in Pten cKO BMSCs (Figure 1H). The Pi3k/Akt target gene *Glut1* was not differently expressed (Figure 1I). In BMSCs derived from female mice, there was also a significant increase of *Osx1* and *Col1a1* in Pten cKO compared to controls (Supplemental Figure S3A–F). Cre expression was elevated in all Pten cKO, but not in any control BMSCs (Figure 1J).

To determine the fate of Osx-positive cells targeted by recombinase events, we crossed Pten cKO mice with mTmG reporter mice and analyzed EGFP expression in femora by immunohistochemistry. Predominantly, the bone linings were positive for EGFP, but not the bone marrow, indicating that cells with positive recombination events mainly developed into osteoblasts (Figure 1K).

### Pten cKO in osteoprogenitor cells results in changes in bone architecture

**Figure 2:**
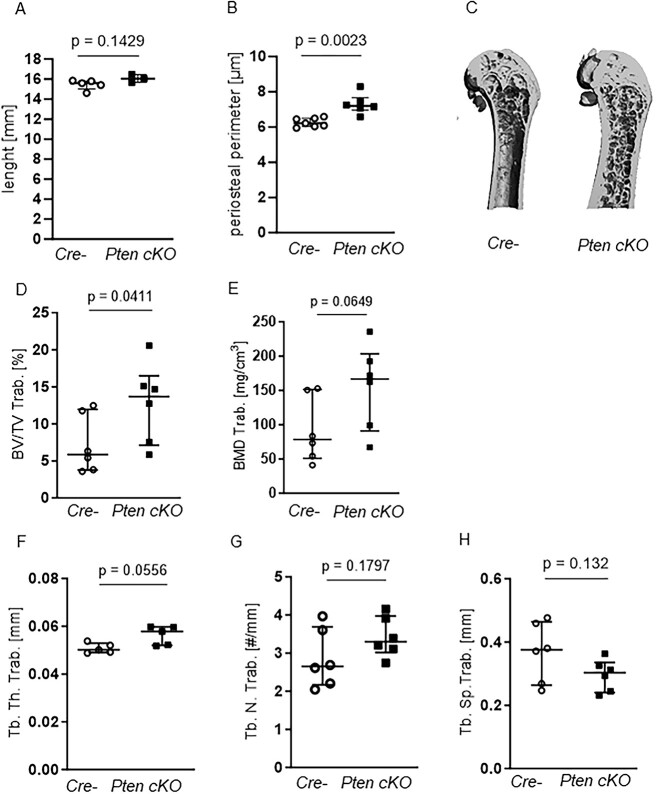
Pten cKO in osteoprogenitor cells results in changes in trabecular bone architecture. (A) Femur length did not differ between Cre-negative (Cre-) and Pten cKO mice, while (B) periosteal perimeter was significantly increased in Pten cKO femora (*P* = .0023, *n* = 7 Cre-, *n* = 6 Pten cKO). The trabecular part of femora was assessed using μCT. (C) A representative image of μCT 3D reconstruction of the distal trabecular femur of Cre- and Pten cKO mice. (D) BV/TV (*P* = .0411, *n* = 6 per group) and (E) BMD (*P* = .0649, *n* = 6 per group) were increased in Pten cKO bones. (F) Femoral trabecular thickness (Tb.Th) was higher in Pten cKO femora (*P* = .0556, *n* = 6 per group), with a trend toward (G) higher trabecular number (*P* = .1797) and (H) less separation (*P* = .132).

Next, we checked whether the increased Pi3k signaling in osteoprogenitor cells is associated with alterations in bone architecture. Longitudinal bone growth was not affected by Pten deletion in osteoprogenitor cells (Figure 2A), but periosteal growth indicated by femoral periosteal circumference was altered in Pten cKO mice (Figure 2B and C  *P* = .0023, *n* = 6). At 10 wk of age, the trabecular bone volume in the femura of Pten cKO mice was not different compared to their littermate controls (data not shown). At 38-42 wk of age, Pten cKO mice demonstrated a higher proportion of trabecular bone volume (BV/TV, *P* = .0411, Figure 2D) and a higher BMD (*P* = .0649, *n* = 6, Figure 2E) compared to Cre-negative mice). At the structural level, Pten cKO bones had a higher trabecular thickness (Tb. Th. Trab., *P* = .0556, *n* = 5, Figure 2F) and a trend to an increased trabecular number (Tb.N, Figure 2G) and a lower trabecular separation (Tb.Sp, Figure 2H) compared to Cre-negative bones. We did not observe any sex-specific differences (Supplemental Figure S4A–E) in the trabecular bone parameters.

**Figure 3:**
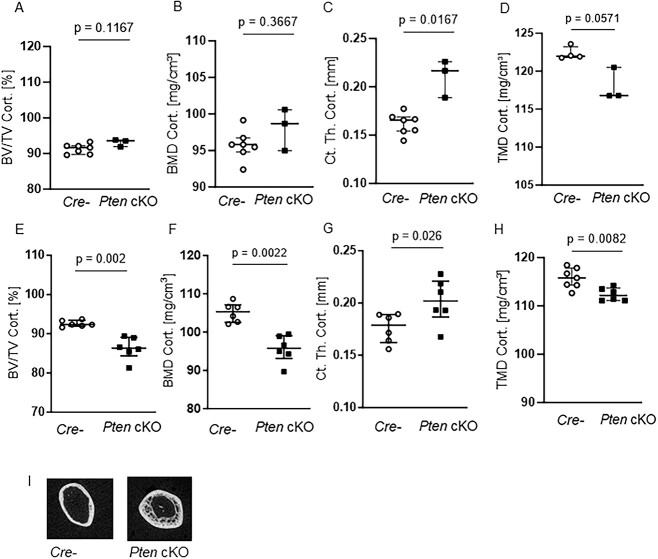
Pten cKO in osteoprogenitor cells results in progressive changes in cortical bone architecture. The cortical part of femora of 10-wk-old (A–D) and 38- to 42-wk-old (E–H) Cre-negative (Cre-) and Pten cKO mice was assessed using μCT: although (A) cortical BV/TV and (B) cortical BMD were not different, (C) cortical thickness (Ct. Th. Cort.) was increased (*P* = .0167, *n* = 7 Cre-, *n* = 3 Pten cKO) and (D) cortical tissue mineral density (TMD) was lower (*P* = .0571, *n* = 4 Cre-, *n* = 3 Pten cKO) already in femora of 10-wk-old mice. In 38- to 42-wk-old mice, (E) cortical BV/TV (*P* = .002, *n* = 6 per group), (F) cortical BMD (*P* = .0022, *n* = 6 per group), and (H) cortical TMD (*P* = .0022, *n* = 7 Cre-, *n* = 6 Pten cKO) were lower in Pten cKO femora compared to controls. (G) Cortical thickness was increased (*P* = .026, *n* = 6 per group). (I) Representative image of 3D reconstruction of the distal cortical femur of 38- to 42-wk-old Cre- and Pten cKO mice. Data are presented as median with interquartile range.

Micro-CT imaging of 10-wk-old mice showed that, although cortical BV/TV (Figure 3A) and BMD (Figure 3B) were unchanged, cortical thickness was significantly higher (Ct. Th.Cort., *P* = .0167, *n* = 7 Cre negatives, *n* = 3 Pten cKO, Figure 3C) and tissue mineral density tended to be lower (TMD Cort., *P* = .0571, *n* = 4 Cre negatives, *n* = 3 Pten cKO, Figure 3D) in Pten cKO mice. In contrast, at 38–42 wk of age, cortical BV/TV (*P* = .002, *n* = 6 per group, Figure 3E) and BMD (*P* = .0022, *n* = 6, Figure 3F) were significantly lower in Pten cKO bones. On the other side, the phenotype seen in 10-wk-old mouse femora regarding cortical thickness (*P* = .026, Figure 3G) and tissue mineral density (*P* = .0082, Figure 3H) was more exacerbated. The same trends were seen in female mice (Supplemental Figure S4F–H). The representative images of 38- to 42-wk-old Pten cKO mice show the altered cortical structure. It was very difficult to distinguish between trabecular and cortical bone, and it seems that the trabecular bone was extending into the diaphysis (Figures 2C and 3I).

### Pten cKO mice increased bone turnover and altered bone remodeling

**Figure 4:**
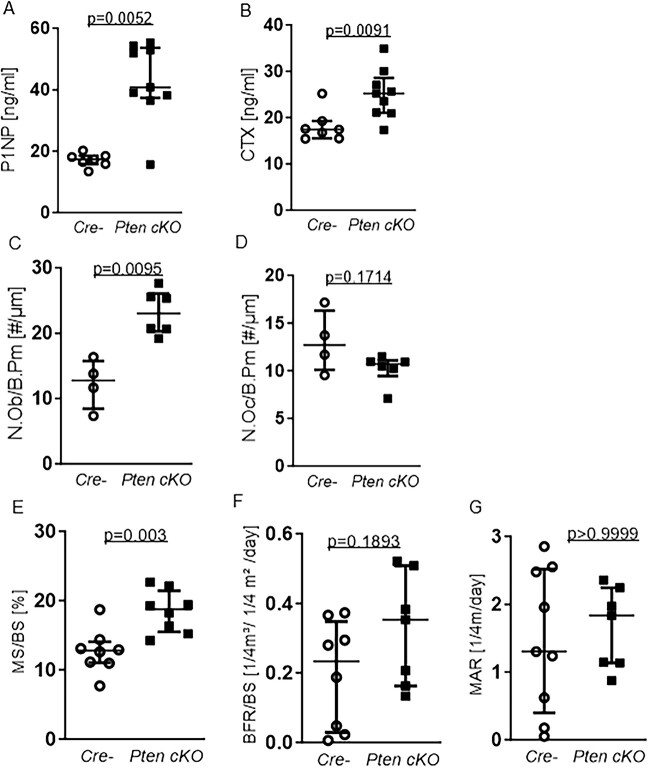
Pten cKO osteoprogenitor cells increases bone turnover and influences bone remodeling. Serum markers for bone turnover were quantified using ELISA. (A) Serum P1NP as a marker for bone formation. P1NP was elevated (*P* = .0052, *n* = 7 Cre negative (Cre-), *n* = 9 Pten cKO). (B) C-terminal telopeptide of type 1 collagen (CTX) as a marker for bone resorption was increased (*P* = .0091, *n* = 7 Cre-, *n* = 9 Pten cKO). (C) Number of osteoblasts/bone perimeter (N.Ob/B.Pm) was increased (*P* = .0095, *n* = 4 Cre-, *n* = 6 Pten cKO) in bones from Pten cKO mice compared with controls. (D) Number of osteoclasts/bone perimeter (N.Oc/B.Pm) was not significantly changed (*n* = 4 Cre-, *n* = 6 Pten cKO). (E) Trabecular mineralizing surface per bone surface (MS/BS) was higher in Pten cKO femora (*P* = .003, *n* = 8 per group) while (F) there was a trend toward higher BFR/BS (, *P* = .1893, *n* = 8 Cre-, *n* = 7 Pten cKO). Data are presented as median with interquartile range.

High bone mass is either a result of increased bone formation or impaired bone resorption. As the trabecular and cortical bone alterations were more pronounced in older mice, we assessed bone turnover in 38- to 42-wk-old Pten cKO mice by performing serum analysis of bone turnover markers and dynamic bone histomorphometry. Serum P1NP as a marker for bone formation was 2-fold higher in Pten-deficient mice in comparison to controls (*P* = .0052, *n* = 7 Cre negatives, *n* = 9 *Pten* cKO) (Figure 4A). The bone resorption marker C-terminal telopeptide of type 1 collagen (CTX) was also increased in the serum of Pten cKO mice (*P* = .0091, *n* = 7 Cre negatives, *n* = 9 *Pten* cKO, Figure 4B). In line with the increase of P1NP, we observed an increased number of osteoblasts per bone perimeter (N.Ob/B.Pm) (*P* = .0095, *n* = 4 Cre-negatives, *n* = 6 *Pten* cKO, Figure 4C) in trabecular bones of *Pten* cKO mice compared to control mice. The number of osteoclasts was not altered (*n* = 4 Cre negatives, *n* = 6 *Pten* cKO, Figure 4D). The analysis of female mice showed similar results (Supplemental Figure S5A–D).

In line with the increased number of osteoblasts, dynamic bone histomorphometry in the trabecular bone revealed a significantly higher mineralizing surface to bone surface ratio (MS/BS) in 38- to 42-wk-old male Pten cKO mice (*P* = .003, *n* = 8 per group, Figure 4E) and a trend toward a higher bone formation rate/bone surface (BFR/BS, *P* = .1893, *n* = 8 per group, Figure 4F), while mineral apposition rate (MAR) was not altered (Figure 4G).

### Bones from Pten cKO mice display a higher elasticity and mechanical stability

**Figure 5:**
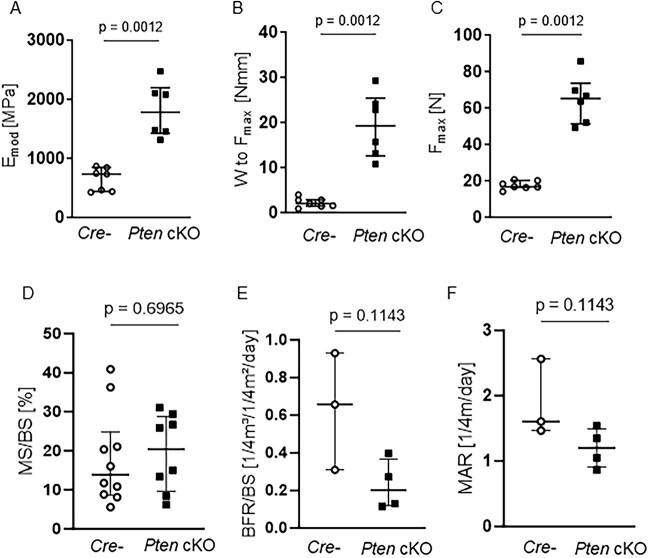
Pten cKO increases bone stability and mechanical strength without altering cortical bone remodeling. Biomechanical properties at the femoral shaft were assessed by three-point bending test. Pten cKO femora (*n* = 6) showed increased mechanical strength compared to femora of Cre-negative (Cre-*n* = 7) mice with (A) increased elastic modulus (Emod, *P* = .0012), (B) significantly increased work-to-fracture (W to F_max_, *P* = .0012) and (C) significantly higher load or maximum force (F_max_, *P* = .0012). (D) Cortical mineralizing surface per bone surface (MS/BS), (E) cortical bone formation rate, and (F) cortical marrow apposition rate were not different. Data are presented as median with interquartile range.

When handling bones, we observed that the tibia and femur of Pten cKO mice were paler (Supplemental Figure S6A) than Cre-negative bones, which was confirmed by measuring the integrated density of Pten cKO and Cre-negative bones (Supplemental Figure S6B). The alterations we observed in cortical bone of 38- to 42-wk-old Pten cKO mice suggest alterations in bone strength. Just by handling the bones, we noticed a striking difference in bone stability. Pten cKO femora and tibiae were less prone to break than bones of Cre-negative mice. To quantify this observation, we assessed the maximum force at the femoral shaft by three-point bending analysis (*n* = 7 Cre negatives, *n* = 6 Pten cKO). Pten cKO showed improved mechanical strength with increased elastic modulus (E_mod_, *P* = .0012, Figure 5A), significantly increased work-to-fracture (W to F_max_, *P* = .0012, Figure 5B), and significantly higher maximum force (F_max_, *P* = .0012, Figure 5C) of Pten cKO femora compared to controls. Results were similar in female mice (Supplemental Figure S6C–E). As the reduced consolidation of cortical bone reflected by a lower cortical BMD and TMD would rather suggest an impaired bone strength, we performed dynamic bone histomorphometry of the cortical bone to understand the increased resistance to fracture. We did not observe any changes in the mineralizing surface, mineral apposition, and bone formation rate between Pten cKO mice and their controls (Figure 5D–F). Tartrate-resistant acid phosphatase (TRAP) staining of cortical bone revealed no significant changes in osteoclast and osteoblast numbers (Supplemental Figure S6F and G). Moreover, we analyzed the amount of nonmineralized bone (osteoid) in the cortical bone by von Kossa/van Gieson staining and found no differences in osteoid width (O.Wi), osteoid volume (OV), and osteoid surface (OS) between Pten cKO and control mouse bone (Supplemental Figure S6H–J).

To check whether an increased production of matrix proteins would potentially contribute to the observed higher elasticity and stability of Pten cKO bones, we assessed expression of bone matrix proteins Collagen1A1 and Fibronectin in bones of young (10-wk-old) Pten cKO and Cre-negative mice. Unexpectedly, we saw lower Collagen1A1 protein in whole-bone lysates from Pten cKO mice (*n* = 6 Cre negatives, *n* = 5 Pten cKO, *P* = .002, Supplemental Figure S7A), while there was no difference in Fibronectin protein (Supplemental Figure S7B). We then asked whether the Pi3k pathway activation observed in older Pten cKO mouse bones would be already present and found that both Pten downregulation and higher AKT activation could be observed in bones from young mice. Ribosomal S6 kinase, a Pi3k downstream target, and regulator of protein synthesis was, however, lower expressed in Pten cKO bones (*n* = 6 per group, *P* = .0037, Supplemental Figure S7C), which could indicate a slower rate of protein synthesis in Pten cKO bones. To test this, we performed a puromycin incorporation SUnSET (SUrface SEnsing of Translation) assay,^11^ comparing the amount of nascently formed polypeptide chains in BMSCs from Cre-negative and Pten cKO mouse bones after overnight inhibition of protein biosynthesis. In line with lower S6 kinase protein levels, we found lower puromycin-positive proteins in BMSCs from Pten cKO mouse bones compared to Cre negative (Supplemental Figure S7D).

### BMSCs from PKO mice have increased potential for proliferation and osteogenic differentiation

**Figure 6:**
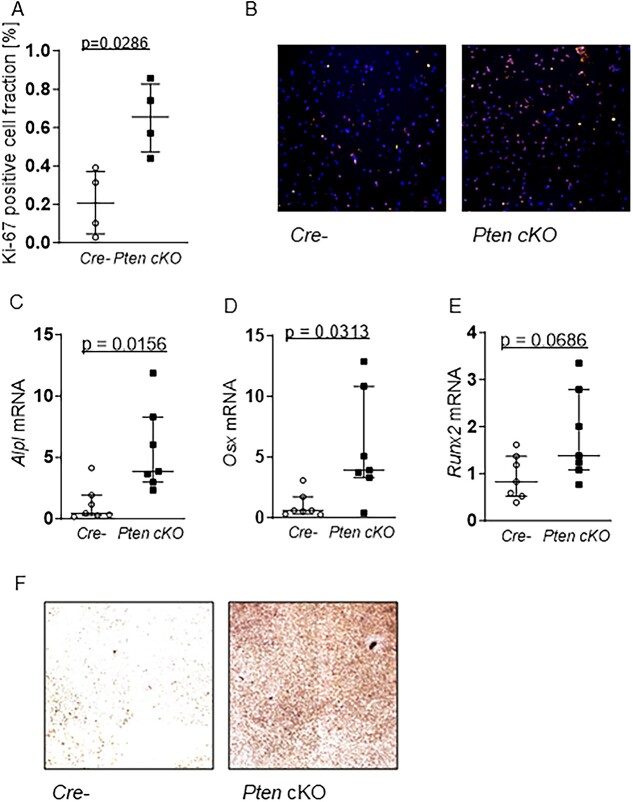
Pten cKO enhances proliferation and capacity for osteogenesis in BMSCs. (A) Proliferation marker Ki-67 immunofluorescence staining (Ki67/Hoechst = proliferative fraction in %) in Cre-negative (Cre-) and Pten cKO BMSCs at day 1 of proliferation: Pten cKO cells show an increase in the fraction of Ki-67–positive cells (*n* = 4, *P* = .0286). (B) Representative images of Ki-67 (red) and Hoechst (blue) staining of Cre- (left) and Pten cKO (right) BMSCs (100× magnification). Cre- or Pten cKO BMSCs (*n* = 7 per group) were differentiated for 14 days in osteogenic differentiation medium. Expression of osteogenesis markers (C) *Alpl* (*P* = .0156), (D) *Osx* (*P* = .0313), and (E) *Runx2* (*P* = .0686), determined using qPCR, was increased at mRNA level in Pten cKO BMSCS. (F) Alkaline phosphatase activity was visualized by hydrolysis of *p*-nitrophenylphosphate staining. Representative images of *n* = 4 independent experiments (40× magnification). Data are presented as median with interquartile range.

Since increased Akt activation can influence cellular responses such as proliferation and differentiation, we determined the proliferative fraction of Pten cKO compared to control BMSCs. Male Pten cKO BMSCs showed an increased fraction of Ki-67-positive cells (*n* = 4, *P* = .0286, Figure 6A and B), indicating increased proliferation. A similar result was seen in BMSCs from female Pten cKO mice compared to controls (*n* = 4, *P* = .0286, Supplemental Figure S8A).

To investigate whether Pten deletion has an impact on osteogenic differentiation capacity in vitro, we compared osteogenic gene expression in control and Pten cKO BMSCs after 14 days of culture in osteogenic differentiation medium. Expression of bone markers *alkaline phosphatase* (*n* = 7, *P* = .0156) and transcription factors *Osx* (*n* = 7, *P* = .0313) and *Runx2* (*n* = 8 Cre negatives and 7 Pten cKO, *P* = .0469) were increased at mRNA level in Pten cKO BMSCs (Figure 6C–E). Osteoblast mineralization as measured by Alp staining after 14 days in an osteogenic differentiation medium was significantly enhanced in Pten cKO BMSCs (*n* = 4 independent experiments, Figure 6F). In contrast, adipogenic differentiation as shown by evaluation of the gene expression of the markers adiponectin, leptin, and peroxisome proliferator-activated receptor (Pparγ) as well as by Nile red staining of lipids was not significantly different between Pten cKO and control BMSCs (Supplemental Figure S8B–E).

## Discussion

In this study, we aimed to determine the impact of persistent Pi3k signaling on bone structure and bone stability by deleting Pten specifically in immature osteoprogenitor cells. Pten phosphatase negatively regulates Pi3K signaling. PI3K and its downstream activation of the Akt pathway increase cell proliferation and survival and thereby also influence bone development^12^. We found that Pten loss in osteoprogenitor cells significantly contributes to trabecular bone architecture and mechanical bone stability in a sex-independent manner by affecting osteogenic differentiation capacity.

Several studies described an impact on skeletal development in association with Pten deletion using different Cre lines. The studies examined Pten deletion at different time points. ﻿Conditional deletion of Pten in Col2a1-expressing chondrocytes led to an increased stress response, disrupted chondrocyte differentiation, and increased bone formation^13–15^. Also Pten deletion in mature osteoblasts using Osteocalcin (Oc)*-*Cre transgenic mice led to increased proliferation and higher bone mass^16^, while deleting Pten in leptin receptor (LepR)–positive osteoprogenitors led to accelerated osteogenic differentiation with a decrease in cell death^16^. Similarly, the deletion of Pten in undifferentiated mesenchyme using Twist family basic helix loop helix transcription factor 2 (Twist2/Dermo)-Cre led to an increased number of osteoblasts and an expanded bone matrix^17^. Vice versa, whole-body deletion of Akt1 and Akt2 as downstream effectors of Pi3k/Pten signaling caused a delay in bone development and ossification^18^.

In temporal order, Dermo-Cre^17^ and LepR-Cre^19^ act earlier and thus more nonspecifically on the cell lineages, while Oc-Cre^16^ acts specifically on the later stages of osteogenic cells.

In our study, we used the Osx-Cre recombinase to downregulate Pten from early critical stages of osteoblast differentiation. Osx is a transcription factor essential for embryonic osteoblast differentiation and also plays a critical role in the formation and function of postnatal osteoblasts that differentiate further and produce matrix proteins, including the main bone constituent, type I collagen ^20^^,^^21^. Although Cre drivers such as Dermo or LepR are more broadly expressed, Osx is mainly expressed in osteoprogenitor cells, osteoblasts, and osteocytes. To reduce Osx-Cre targeting of nonosteoblastic cells ^9^, we used the tetracycline-controlled Tet-Off regulation of Osx-Cre until the age of 4 wk and found Osx-Cre–positive recombination events predominantly in bone lining cells. Osx was also described to target hypertrophic chondrocytes, but interestingly, Pten deletion did not affect longitudinal bone growth.

Our results show that Pten deletion in Osx-positive cells results in increased trabecular bone volume with increased trabecular thickness, a tendency to higher number of trabecular numbers and less trabecular spacing. Pten deletion led to an enhanced bone turnover that was mainly driven by increased osteoblast activity reflected by increased numbers of osteoblasts and a higher bone formation rate. As the mineralized surface was more significantly affected than the MAR, the high bone formation is more a result of enhanced recruitment of osteoblasts than increased osteoblast activity. The trabecular bone phenotype is in line with the study of Liu and colleagues using Pten Oc-Cre mice. They also reported a high trabecular bone mass due to increased bone formation and osteoblast numbers^16^. Another study using also Osx-Cre to study the function of Pten in osteosarcoma development also showed a high trabecular bone volume in mice with a Pten deletion in osteogenic cells (8). Although the calvaria showed a significant thickening, the cortical thickness of femora measured by Filtz et al. remained unchanged compared with the control group. In our study, the cortical thickness in the femur was strongly increased, whereas the cortical BMD as well as TMD were significantly lower. Cortical thickness was also found to be significantly higher in long bones of Oc-Cre Pten KO mice ^14^. While the other studies did not analyze the cortical bone in more detail, we further explored the role of Pten in cortical bone. Despite the lower BMD of the cortical bone, Pten cKO bones were more resistant to fracture and displayed a higher elasticity. As the TMD was also reduced in Pten cKO mice, we analyzed if mineralization and bone formation would be affected in the cortical bone, which would explain the stability of Pten cKO mice. However, we did not find any differences in cortical bone formation. The content of nonmineralized bone (osteoid) was also not different between Pten cKO mice and controls. Increased bone resorption could not explain the lower BMD and TMD in the cortical bone as osteoclast numbers were not changed. Additionally, we could rule out that increased production of bone matrix proteins collagen 1A1 or fibronectin contributed to the observed bone phenotype. Interestingly, we found less puromycin incorporation into newly synthesized polypeptide chains in Pten cKO BMSC protein lysates compared to controls. This finding was supported by detecting lower total levels of the mTOR target ribosomal protein S6 kinase in BMSCs from Pten cKO mice, while, as expected, AKT phosphorylation was increased, pointing to a downregulation of mTOR-dependent protein synthesis. In summary, most likely, the increased and extended trabecular bone volume stabilizes Pten cKO bones, resulting in greater resistance to fracture despite lower cortical BMD and TMD.

However, it remains speculative if Pten regulates the process of cortical bone formation in another manner. *Osx*-positive cells were shown to be important for corticalization, the consolidation of trabecular bone into the metaphyseal cortex ^22^. A loss of function of Pten in these cells might accordingly delay corticalization leading to an increased cortical trabecular thickness due to trabecular bone extending in the diaphysis, but to a low cortical BMD. In addition, also osteocytes express Osx and could be affected by Pten deletion. An osteocyte-specific Pten deletion would help to explain the function of Pten in osteocytes and their consequences on bone stability.

On the cellular level, we found that BMSCs derived from Pten cKO mice and subjected to osteogenic and adipogenic differentiation conditions in vitro showed a striking increase in osteogenesis compared to Cre-negative mice. Pluripotent mesenchymal stem cells expressing Osx can not only differentiate into osteoblasts and chondrocytes but also into bone marrow adipocytes, depending on the type of their transcriptional differentiation program^9^^,^^23^. Increased capacity of BMSCs for osteogenesis was found regardless of which Cre system was used to restrict Pten function^8^^,^^16^^,^^17^^,^^19^. Proliferative capacity was similarly increased in Pten cKO BMSCs, pointing to either greatly increased Osx-Cre targeted cell numbers during cultivation or possibly the secretion of growth factors from Pten cKO BMSCs that act on Pten wild-type BMSCs, enhancing their proliferation and osteogenic capacity.

Osx transcript and Osx protein were found to be expressed early during hematopoiesis, in subsets of hematopoietic stem cells and multipotent progenitor populations^24^. In line with these findings, we noticed that Pten cKO mice had a disturbed hematopoiesis, as shown macroscopically by the paleness of Pten cKO distal femora and tibiae compared to controls. The increased spleen size could point to extramedullary hematopoiesis, and a difference in blood cell count (higher lymphocytes) would also occur in hematological diseases, so we cannot fully rule out this possibility.

Although we used the Tet-off regulation of Osx activity, we cannot completely rule out that Cre could also be expressed earlier in mouse pups that do not take up adequate amounts of doxycycline-containing milk. This would presumably lead to Pten knockout in the perinatal and adult mesenchymal precursor cell population residing in the bone marrow as well as in osteoblasts and osteocytes in our mouse model^9^^,^^25^. Our finding of predominantly targeted bone lining cells in our Pten cKO mouse model speaks against a major Pten loss in the bone marrow, although we could detect reduced Pten and increased Akt phosphorylation both in whole-bone protein lysates and in cultured BMSCs isolated from Pten cKO mice compared to controls. In conclusion, conditional Pten knockout in Osx-expressing preosteoblasts leads to alterations in trabecular and cortical bone and an increased bone stability and elasticity. These results indicate that Pten knockout in preosteoblasts impacts bone function via reduced counter-regulation of the growth-promoting Pi3k signaling cascade and suggests changes in bone turnover and strength. Pten deletion in osteoprogenitor cells increased osteoblast proliferation and differentiation capacity pointing to the potential of using Pten inhibitors for treating conditions related to bone fragility or impaired bone formation. A similar approach has been followed by using adiponectin to downregulate PTEN expression, which was shown to promote the osteogenic differentiation of human BMSCs^26^. The next step could be testing Pten pharmacologic inhibition in models of impaired bone stability and osteoporosis.

## Materials and methods

### Material

Cell culture media and supplements were obtained from Gibco or Invitrogen. Unless otherwise stated, chemicals were bought from Sigma (Sigma-Aldrich).

### Animals

Breeding and phenotyping of mice were carried out according to the guidelines approved by the local authorities of the State of Saxony, Germany, as recommended by the responsible local animal ethics review board (Regierungspräsidium [TVV30/19 and TVV32/17]). Mice with loxp sites flanking Pten exon 5 (Stock B6.129S4-Ptentm1Hwu/J, 006440, Jackson labs)^27^ were crossed with mice transgenic for a Tet-off GFP::Cre fusion construct under the transcriptional regulation of the Osx1 promoter (B6.Cg-Tg(Sp7-tTA,tetO-EGFP/cre)1Amc/J;^28^), kindly provided by Dr Stuart H. Orkin, Boston Children’s Hospital^29^. For determining fate of cells targeted by Cre recombinase, mice were intercrossed with mTmG mice (B6.129(Cg)-Gt(ROSA)26Sortm4(ACTB-tdTomato,-EGFP)Luo/J), kindly provided by Prof Tim J. Schulz, DIfE (German Institute of Human Nutrition). Cre expression in breeding mice was suppressed by 625 mg/kg doxycycline in feed (A112D70624, Ssniff Spezialdiäten). Mice included in experiments were fed with normal chow after weaning (at 3 wk) for Cre induction. Cre-negative mice served as controls. Genotyping for Pten loxp sites and Cre transgene expression was carried out as described^27^. Respective primer sequences are listed in Table 1. Blood was collected by cardiac puncture into K EDTA containers. Blood count analysis was performed by Synlab.vet GmbH.

**Table 1:** Genotyping primers.

**Primer**	**Sequence**
mPten_loxp_genotyp.oIMR9554_FW	CAAGCACTCTGCGAACTGAG
mPten_loxp_genotyp.oIMR9555_RV	AAGTTTTTGAAGGCAAGATGC
mOsx_Cre_FW	GACCAGGTTCGTTCACTCATGG
mOsx_Cre_RV	AGGCTAAGTGCCTTCTCTACAC
mPten_exon5_P1	ACTCAAGGCAGGGATGAGC
mPten_exon5_P2	AATCTAGGGCCTCTTGTGCC
mPten_exon5_P3	GCTTGATATCGAATTCCTGCAGC

### Sample collection and cell culture of stromal cells from mouse bone

Mice were sacrificed by cervical dislocation, and tissues for further analyses were collected. Femora and tibiae were dissected from the surrounding tissues. For further bone analyses, bones were fixed in Roti-Histofix 4% (Carl Roth GmbH, P087) for 72 h. For whole protein analysis, bones were snap frozen at −80 °C. Bone marrow cells were isolated from femora and tibiae of Pten cKO and Cre-negative mice. The epiphyseal growth plates were removed, and the BMSCs were collected by either flushing with PBS in case of collecting the pellets for genomic DNA isolation or flushing with Minimum Essential Medium (Gibco) containing 1% 2 mM glutamine, 1% penicillin–streptomycin, and 15% FBS Superior (Biochrom GmbH; S 0615) with a 27-gauge needle in case of subsequent culturing of BMSCs. Cells were grown at 37 °C in a humidified atmosphere of 95% air and 5% CO_2_. Cell counts were determined with a hemacytometer after trypan blue staining. On day 5, the cells were passaged.

### Pten exon 5 deletion

To check the deletion of Pten exon 5 in the BMSC fraction, we isolated genomic DNA from BMSC pellets using the QiaAmp DNA Mini kit (Qiagen, 51 304) and amplified a section surrounding exon 5 by PCR^27^. PCR products were visualized on a 1.5% agarose gel. A band size of 400 bp indicated knockout of Pten exon 5, while an 1100 bp band occurred after amplification of Pten exon 5 including the flanking loxp sites. Primer sequences are listed in Table 1.

### In vitro *osteogenesis and adipogenesis*

Osteogenic differentiation was induced by using the MesenCult osteogenic stimulatory kit (Stem Cell Technologies, #05504). The medium was changed every 2 days. Alkaline phosphatase (Alp) activity was visualized after 14 days of differentiation as described previously^30^. Briefly, after washing with water, cells were fixed for 30 s with acetone citrate solution (60% acetone, 40% 1:50 diluted citrate solution [Sigma 854C-20 mL]), then washed, and stained by adding 300 μL per well of staining solution (dissolve 1 capsule Fast Violet [Sigma 851-10cap] in 48 mL H_2_O + 2 mL Naphthol AS-mix [Sigma 855-20 mL]). After incubation for 30 min in the dark at room temperature, cells were washed with water, and bright-field images were taken. Expression of *Alp*, *Osx*, and *Runx2* was measured by qPCR after 14 days of differentiation.

Adipogenic differentiation was induced by MesenCult adipogenic stimulatory kit (Stem Cell Technologies, #05507), with medium change every 4 days. Adipogenesis was assessed after 14 days of differentiation by Nile Red stain and quantifying gene expression of adiponectin, leptin, and Pparγ.

### Proliferation

For proliferation assays, cells were seeded at a density of 15 000 cells/well (24 h) on two different 96-well plates. On day 1, cells of one plate were fixed and stored at 4 °C until analyzation. On plate two, the cultivation medium was replaced every 72 h. After 7 days, cells were fixed and nuclei were stained with Hoechst 33342 (Sigma) for 5 min at a concentration of 1 μg/mL in PBS. Hoechst fluorescence was detected at 455 nm.

### Immunofluorescence staining

For GFP staining of bones, tissues were fixed in neutral-buffered formalin overnight and processed for paraffin embedding and sectioning. Sections were blocked in 4% BSA and 0.1% Triton-X/PBS for 1 h at room temperature and stained for 1 h at room temperature with goat anti-GFP (Abcam ab6673, 1:500) in 1% BSA/PBS. Following washing in PBS, sections were stained with AF546 donkey anti-goat (Life technologies A11056, 1:500) for 1 h at room temperature, washed in PBS, and then incubated for 1 min with 4',6-diamidino-2-phenylindole (DAPI) for nuclei staining.

For ki-67 staining, cells were fixed in Roti-Histofix 4% (Carl Roth GmbH) after 7 days of cultivation. Cells were permeabilized and blocked in IF-buffer (PBS + 5% BSA + 0.3% Tween20) for 1 h at room temperature and stained with Ki-67 primary antibody overnight at 4 °C (Table 2). Cells were washed three times with IF-buffer and incubated with a secondary antibody (Invitrogen; Thermo Fisher Scientific, Inc., ) for 2 hours at room temperature in the dark. After washing (2 × IF buffer, 1 × PBS), nuclei were stained with Hoechst33342 (Sigma-Aldrich) for 5 min at a concentration of 1 μg/mL in PBS.

**Table 2:** Antibodies used for Western blot (Wb) and immunofluorescence staining (IF).

**Primary antibody**	**Dilution**	**Supplier**	**Cat. No.**
PTEN (138G6) Rabbit mAb	1:1000 TBS-T 5%BSA (Wb)	Cell Signaling Technology	#9559
AKT antibody Rabbit polyclonal Ab	1:1000 TBS-T 5%BSA (Wb)	Cell Signaling Technology	#4058
Phospho-AKT (S473) Rabbit mAb	1:1000 TBS-T 5%BSA (Wb)	Cell Signaling Technology	#4056
Alpha-Tubulin (11H10) Rabbit mAb	1:1000 TBS-T 5%BSA (Wb)	Cell Signaling Technology	#2125
Col1A1 Rabbit polyclonal Ab	1:500 TBS-T 5% milk (Wb)	Thermo	#PA5-29569
Fibronectin (IST-9) mouse mAb	1:1000 TBS-T 5% milk (Wb)	Santa Cruz BT	Sc-59 826
Puromycin mouse mAb (12D10)	1:10.000 TBS-T 5% milk (Wb)	Sigma	MABE343
Anti-Mo/Rt-Ki-67, eBioScience, Clone:SolA15	1:100 IF-buffer (IF)	Invitrogen	#14-5698-82
Goat anti-GFP polyclonal Ab	1:500 1%BSA/PBS (IF)	Abcam	ab6673
**Secondary antibody**	**Dilution**	**Supplier**	**Cat. No.**
Polyclonal goat anti-rabbit immunoglobulin/HRP	1:2000 TBS-T 5% milk (Wb)	Dako	#P0447
Polyclonal goat anti-mouse immunoglobulin/HRP	1:2000 TBS-T 5% milk (Wb)	Dako	#P0448
Alexa Fluor 555 goat anti-rat IgG H + L	1:200 IF-buffer (IF)	Invitrogen	#A21434
AF546 donkey anti-goat Ab	1:500 1%BSA/PBS (IF)	Invitrogen	# A11056

For lipid staining, cells were fixed in Roti-Histofix 4% (Carl Roth GmbH) after 14 days of cultivation and washed with PBS. Afterward, cells were costained with the fluorescent dyes Nile Red (0.5 μg/mL, Sigma) and Hoechst 33342 (1 μg/mL, Sigma) for 5 min in PBS.

Microscopic images were taken using the EVOS FL Auto 2 Cell Imaging System (Invitrogen; Thermo Fisher Scientific, Inc.). Counting of nuclei (DAPI channel) and Ki-67–positive cells (RFP channel) was performed using Image J 1.41 (National Institutes of Health).

### SUnSET (SUrface SEnsing of translation) assay

Cells were seeded at a density of 500 000 cells per well in 6-well plates and grown for 24 h. Medium then was changed to culture medium containing 100 nM rapamycin (S1039, Selleckchem LLC) for incubation overnight. On the next day, medium was changed to medium containing 100 nM rapamycin +1 μM puromycin (Sigma) or 1 μM puromycin only for 30 min. Cells were then washed three times with ice-cold PBS and plates were frozen at −80 °C until protein isolation. For determining puromycin incorporation, densitometric values of BMSC lysates incubated with rapamycin + puromycin were subtracted from puromycin-only incubated BMSC lysates as background.

### Western blot analysis

Frozen bones (femora and tibiae of one mouse) were homogenized using a liquid nitrogen–precooled mortar and pestle and after addition of 400 μL modified radioimmunoprecipitation assay (RIPA) buffer containing 1 mM sodium orthovanadate (Sigma, Inc.; 450 243), 1 mM sodium fluoride (Carl Roth GmbH; P756), and 1 × cOmplete Protease Inhibitor Cocktail (Roche; 11 697 498 001) ultrasonicated for 4× 30s in an ice-cooled ultrasonic water bath. Cultivated BMSCs were resuspended in 100 μL modified RIPA buffer. After incubation on ice for 20 min and removal of cell debris by centrifugation (15 min, 15 000 ×*g*, 4 °C), protein concentration was determined using Pierce BCA protein assay (Thermo Scientific) and 10 μg protein was loaded on an SDS–PAGE and transferred onto nitrocellulose membranes using a semidry transfer apparatus. Afterward, membranes were blocked in 5% nonfat dry milk in TBS buffer containing 0.1% Tween 20. Primary antibodies used for immunoblotting were anti-Pten, anti-AKT, anti-phosphoAKT, anti-S6K (Cell Signaling), anti-Col1A1 (Thermo Scientific), anti-Fibronectin (Santa Cruz BT), and anti Puromycin (Sigma). Anti-alpha-Tubulin (Cell Signaling Technology, Inc.) was used as a loading control. Appropriate secondary antibodies were purchased from Dako (Agilent Technologies, Inc.), according to Table 2. Detection of proteins was carried out using Luminata Classico Western HRP Substrate (Merck Millipore) or Amersham ECL Prime Western Blotting Detection Reagent (GE Healthcare). ImageJ 1.41 was used for densitometric analysis of Western blots (National Institutes of Health).

### Reverse transcription–quantitative PCR

For transcriptional analysis, we seeded 500 000 cells/cm^2^ in a culture medium. RNA was extracted after one passage of cultivation. RNA was isolated using TRIzol reagent (Invitrogen) for bone tissue or the RNeasy Mini Kit (Qiagen) for cultured bone cells. RNA contaminated with genomic DNA was purified using the DNA-free DNase Treatment and Removal Reagent Kit (Ambion). Afterward, RNA was reverse transcribed into complementary DNA (cDNA). To check reverse transcription, we amplified a section of the housekeeping enzyme *glyceraldehyde 3 phosphate dehydrogenase* (*Gapdh*) by PCR and visualized the PCR products with an agarose gel electrophoresis. cDNA was used for SYBRGreen qPCR in the QuantStudio 3 Real-Time PCR system (Applied Biosystems). Table 3 contains a list of primers used for qPCR assays. Gene expression data were normalized to housekeepers *Tata box binding protein* (*Tbp*) and *Hypoxanthine phosphoribosyl transferase* (*Hprt*) mRNA.

**Table 3:** Primers used for qPCR.

Tbp	GGGTATCTGCTGGCGGTTT	TGAAATAGTGATGCTGGGCACT
Hprt	TCCTCCTCAGACCGCTTTT	CATAACCTGGTTCATCATCGC
Pten	TCCCAGACATGACAGCCATC	TGCTTTGAATCCAAAAACCTTACT
Osx	GTCCTCTCTGCTTGAGGAAGAA	CCCAGGGTTGTTGAGTCCC
Adiponectin	TGACGACACCAAAAGGGCTC	CACAAGTTCCCTTGGGTGGA
Pparγ	GTCACACTCTGACAGGAGCC	CACCGCTTCTTTCAAATCTTGT
Leptin	TTTCACACACGCAGTCGGTA	CACATTTTGGGAAGGCAGGC
Runx2	CCGAGTCATTTAAGGCTGCAA	AGAAGCTTTGCTGACACGGT
Alpl	GAACAGACCCTCCCCACGAG	TGTACCCTGAGATTCGTCCCT

### Serum analysis of bone turnover marker

Serum concentrations of bone turnover markers P1NP and C-terminal telopeptide (CTX) were quantified using enzyme-linked immunosorbent assays according to the manufacturer’s protocols (catalog numbers: AC-33F1 and AC-06F1, IDS).

### μCT-analysis of bone mass and bone microarchitecture

The bone microarchitecture of the distal femur of 38- to 42-wk-old mice was imaged using μCT (vivaCT40, Scanco Medical) postmortem with an X-ray energy of 70 kVp and isotropic voxel size of 10.5 μm (114 mA, 200 ms integration time). Trabecular (Tb) and cortical (Ct) bone parameters were assessed using 100 slices according to the standard protocols from Scanco. The trabecular bone was analyzed in the metaphyseal region extending away from the growth plate, while cortical bone analysis was performed in the diaphyseal region midway between the femoral head and distal condyles.

Results and parameters such as BV/TV, trabecular number (Tb. N), trabecular separation (Tb. Sp), and thickness (Tb. Th), as well as cortical thickness (Ct.Th) and BMD are presented according to ASBMR guidelines.^31^.

### Bone histology and bone histomorphometry

For dynamic bone histomorphometry, 17- and 38-wk-old mice were injected with 100 μL calcein (6 mg/mL) intraperitoneally on days 5 and 2 before sacrificing the animals. After fixation in Roti-Histofix 4% (Carl Roth GmbH) for 48 h, bones were dehydrated in ascending ethanol series. Subsequently, bones were embedded in methyl acrylate and cut into 7 μm sections to assess the fluorescent calcein labels. Sections were analyzed using fluorescence microscopy (Axio Imager M1, Zeiss) to determine the MAR and the BFR/BS using the Osteomeasure software (OsteoMetrics) following international standards.

To quantify osteoclasts and osteoblast numbers, tartrate-resistant acid phosphataseTRAP staining was performed on 2 μm paraffin sections of femura. For this purpose, bones were decalcified after fixation for 7 days in Osteosoft (Merck). Osteoclasts and osteoblasts were quantified in an area of 0.90 mm^2^ and 0.48 mm^2^ in the center of the bones using the Microscope Axio Imager M1 (Carl Zeiss Jena) and Osteomeasure software (OsteoMetrics). Terminology and quantification procedures were conducted according to the guidelines of the Nomenclature Committee of the ASBMR ^31^. To assess the OV, OS, and O.Wi of cortical bone, 4-μm sections were stained with von Kossa/van Gieson. The Osteomeasure software was used to analyze an area of 0.90 mm^2^ for osteoid staining in the cortical bone.

### Biomechanical testing: three-point bending test

For biomechanical testing, femurs were rehydrated in PBS overnight. Three-point bending test (Zwick Roell) was performed by placing the rehydrated femur onto two supports with an intermediate distance of 6 mm. The mechanical force was applied vertically in the middle of the femoral midshaft. The measurement started after reaching a preload of 1 N and was performed with a load rate of 0.05 mm/s until failure. Results of the maximal applicable force (F_max_) as an indicator of cortical bone strength were evaluated using testXpert II—V3.7 software (Zwick Roell).

### Statistical analysis

Data are presented as median with interquartile range using GraphPad Prism 10 software (GraphPad Software, Inc.). Outliers were removed using Grubbs test (α = 0.05). For the comparison of two experimental groups, medians were compared via Mann–Whitney test. To determine the significance of fold changes, we used one sample Wilcoxon tests and compared it to the hypothetical median 1. Exact *P*-values are given, *P* < .05 was considered statistically significant.

## Supplementary Material

231129_Supplementary_Figures_ziad016

231129_Supplemental_Figure_Legends_ziad016

## Data Availability

All data is either supplied within the manuscript and supplementary material or available upon request.
