# Supplementary material for: Pten knockout in mouse preosteoblasts leads to changes in bone turnover and strength
Source: JBMR Plus. 2024 Jan 4;8(3):ziad016. doi: 10.1093/jbmrpl/ziad016 (PMC10945711; doi:10.1093/jbmrpl/ziad016)
Supplement: 231129_Supplementary_Figures_ziad016 [file 231129_supplementary_figures_ziad016.pptx]

## Slide 1
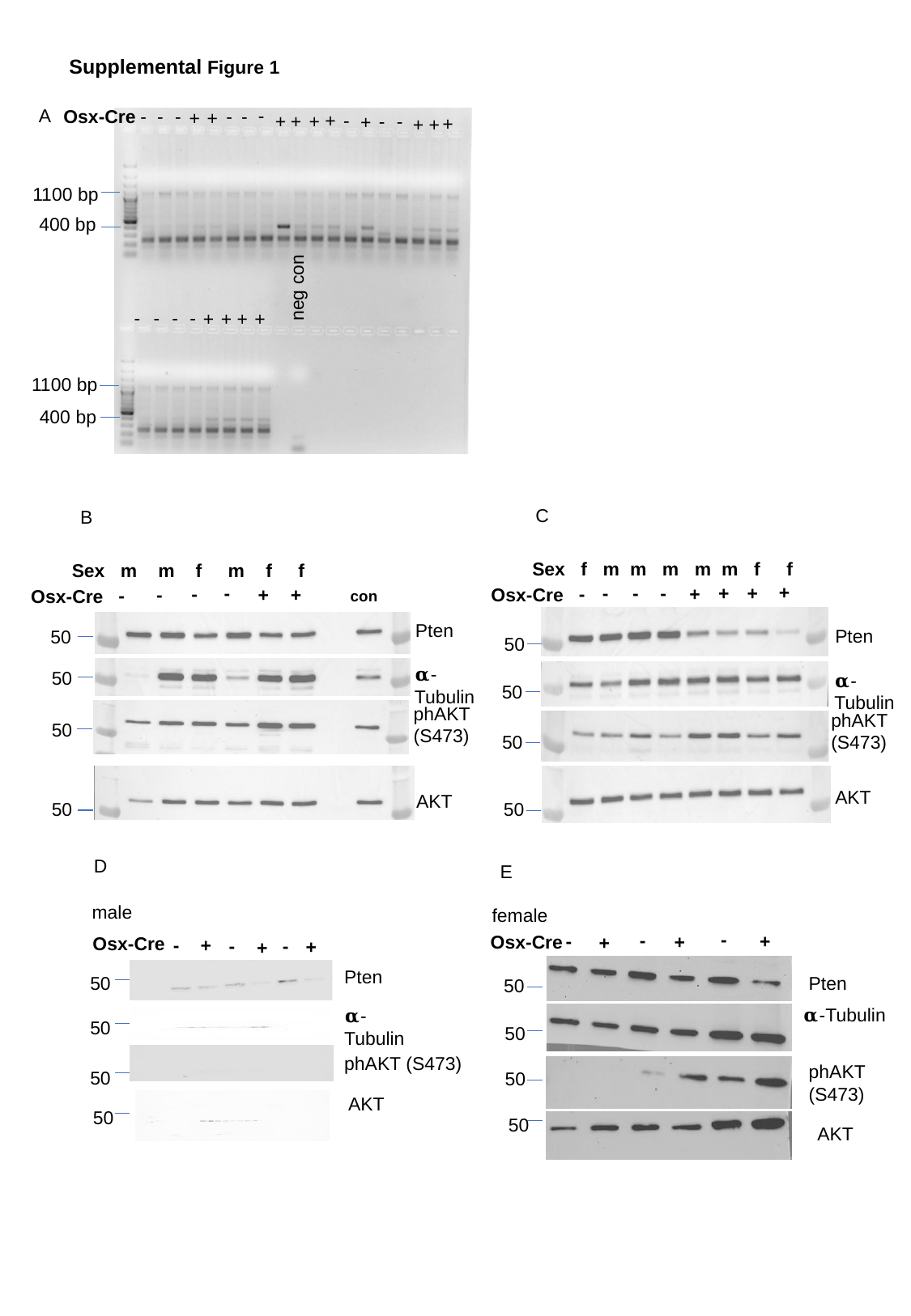

Supplemental Figure 1
A
-
Osx-Cre
-
-
-
-
-
+
+
+
-
+
+
+
-
-
+
+
+
+
1100 bp
400 bp
neg con
-
-
-
-
+
+
+
+
1100 bp
400 bp
C
B
Sex f m m m m m f f
Sex m m f m f f
+
-
+
+
-
-
-
-
+
-
+
+
Osx-Cre
-
-
Osx-Cre
con
Pten
Pten
50
50
𝛂-Tubulin
50
𝛂-Tubulin
50
phAKT
(S473)
phAKT
(S473)
50
50
AKT
AKT
50
50
D
E
male
female
-
-
+
-
Osx-Cre
+
+
Osx-Cre
-
+
-
-
+
+
Pten
Pten
50
50
𝛂-Tubulin
𝛂-Tubulin
50
50
phAKT (S473)
phAKT
(S473)
50
50
AKT
50
50
AKT

## Slide 2
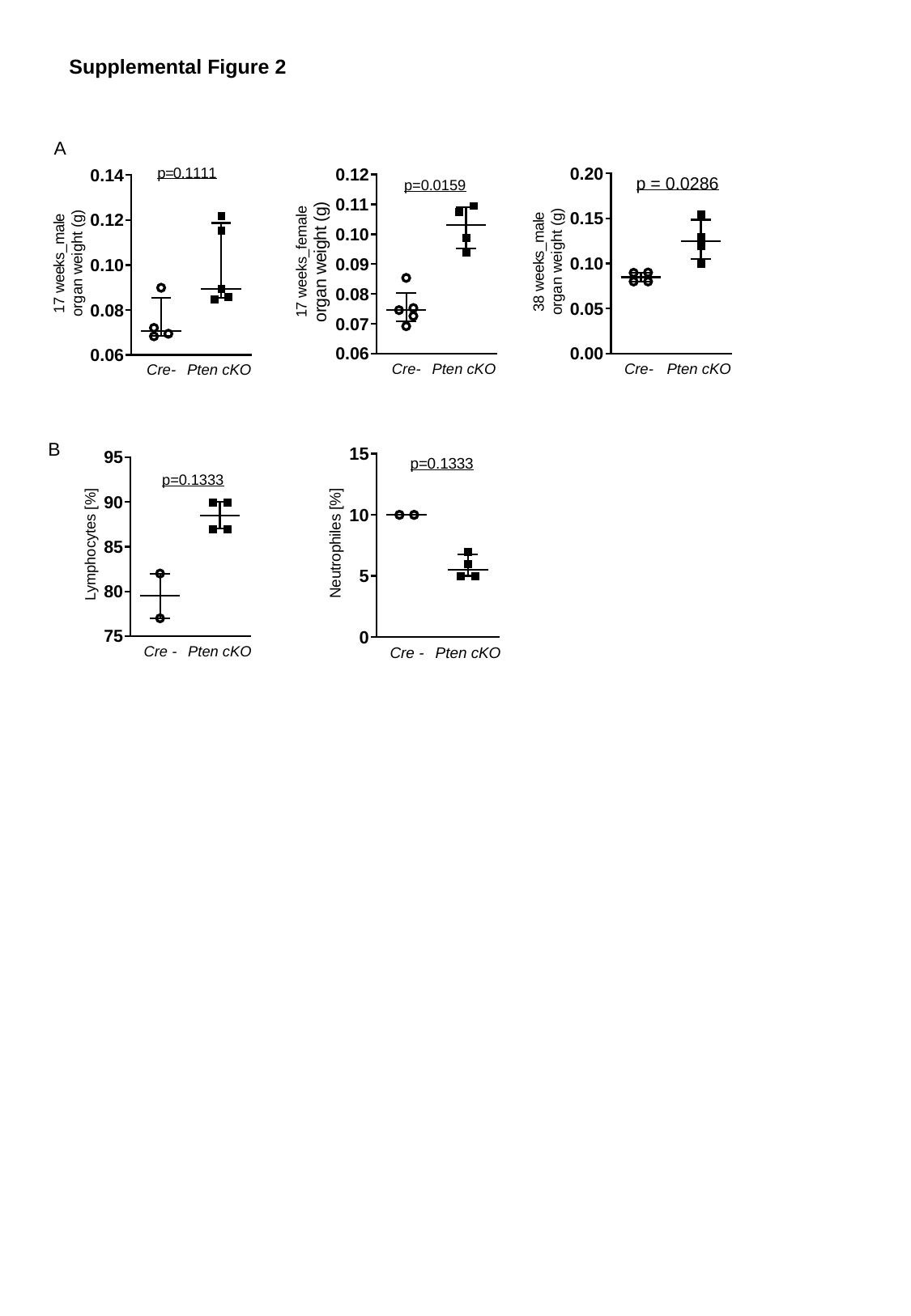

Supplemental Figure 2
A
B

## Slide 3
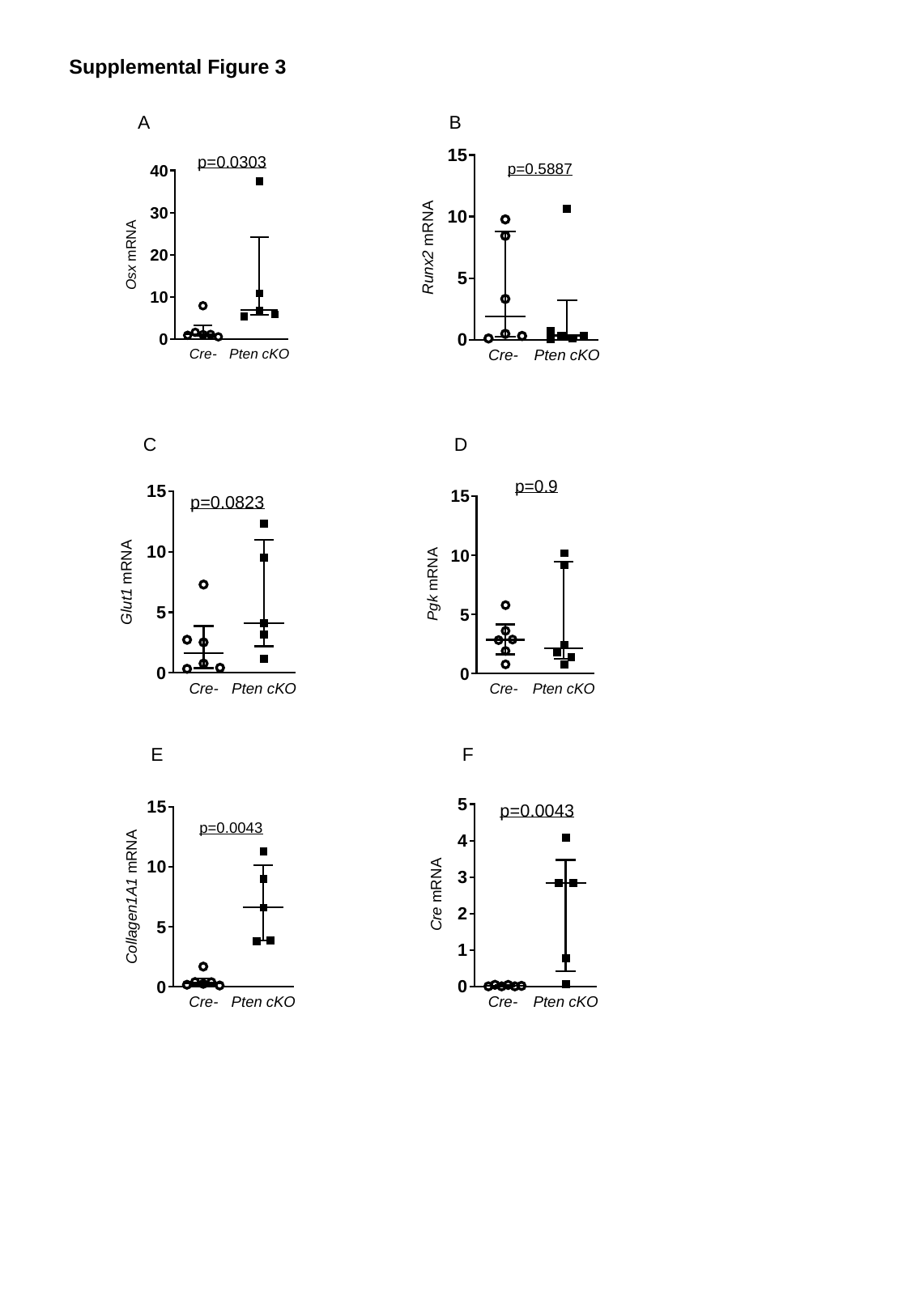

Supplemental Figure 3
A		 B
C		 D
E		 F

## Slide 4
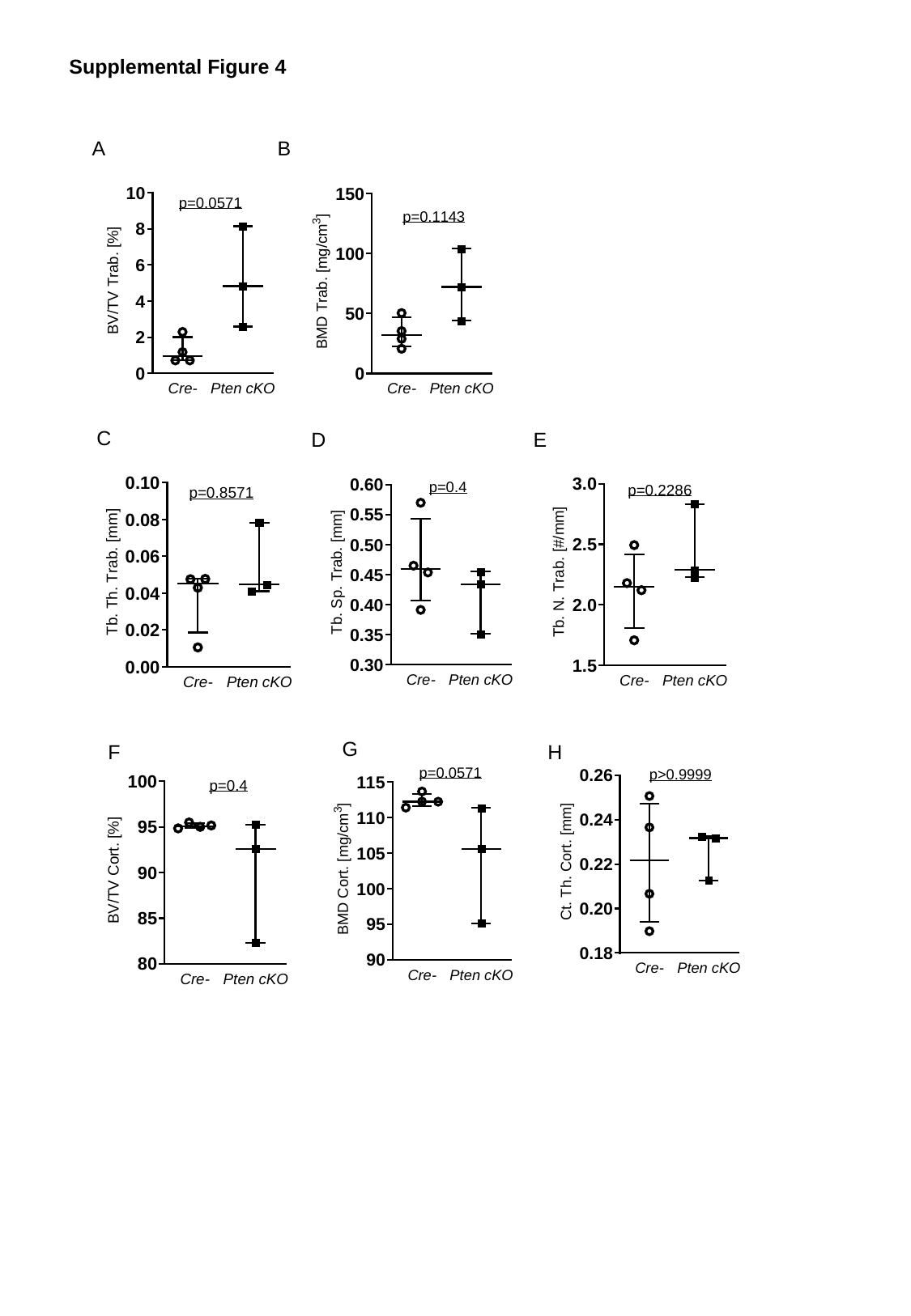

Supplemental Figure 4
B
A
C
E
D
G
H
F

## Slide 5
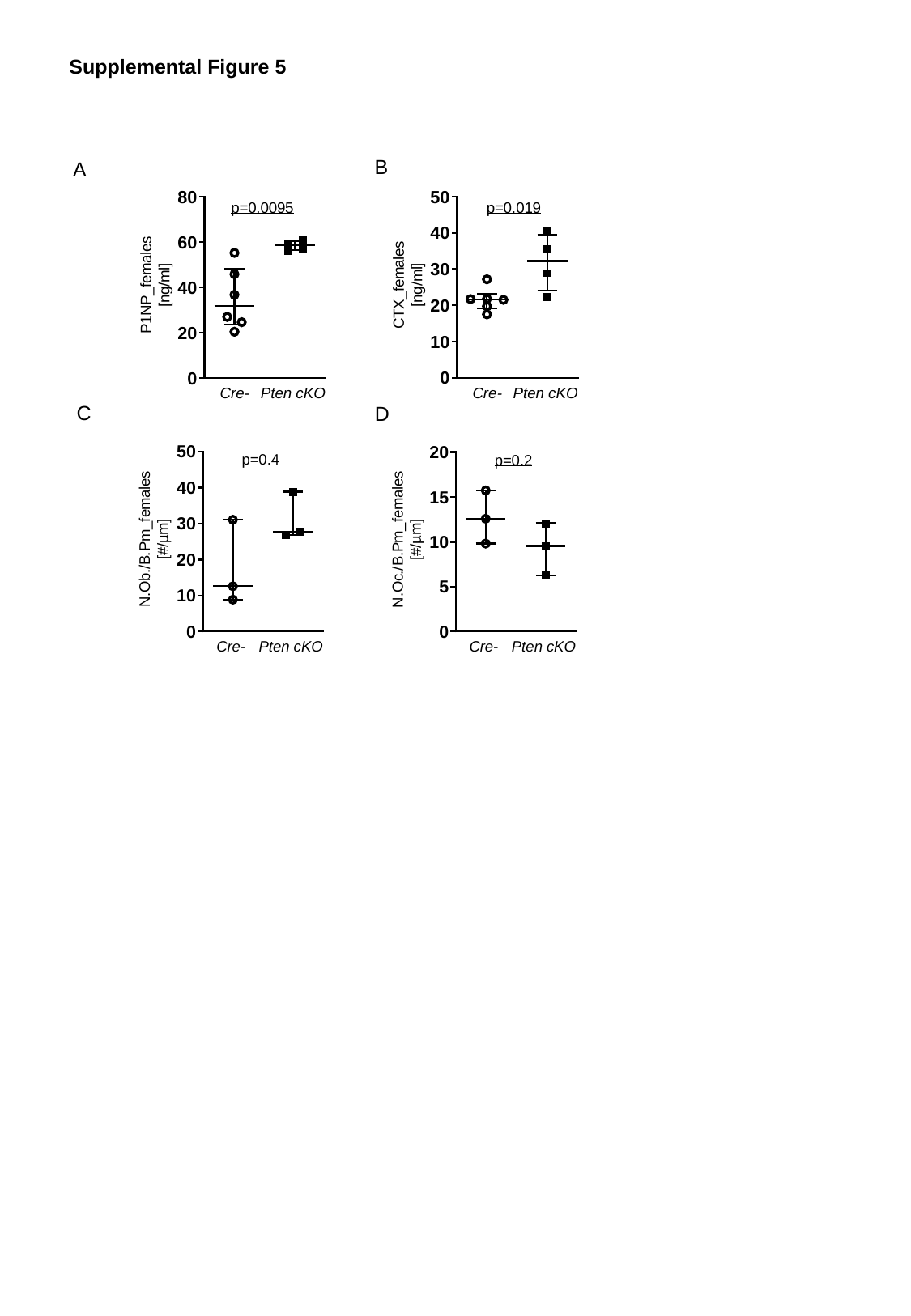

Supplemental Figure 5
B
A
C
D

## Slide 6
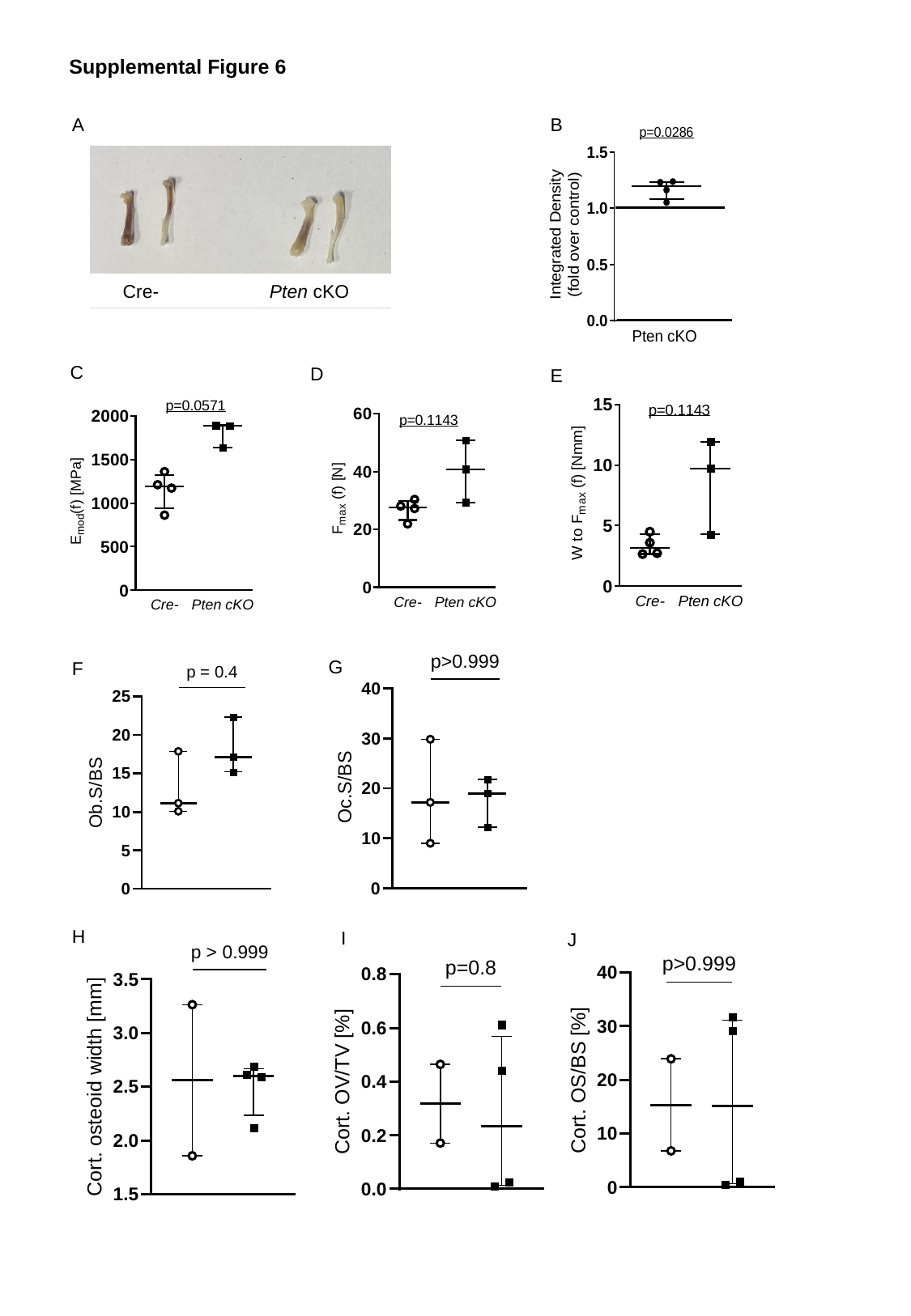

Supplemental Figure 6
A
B
 Cre- Pten cKO
C
D
E
G
F
H
I
J

## Slide 7
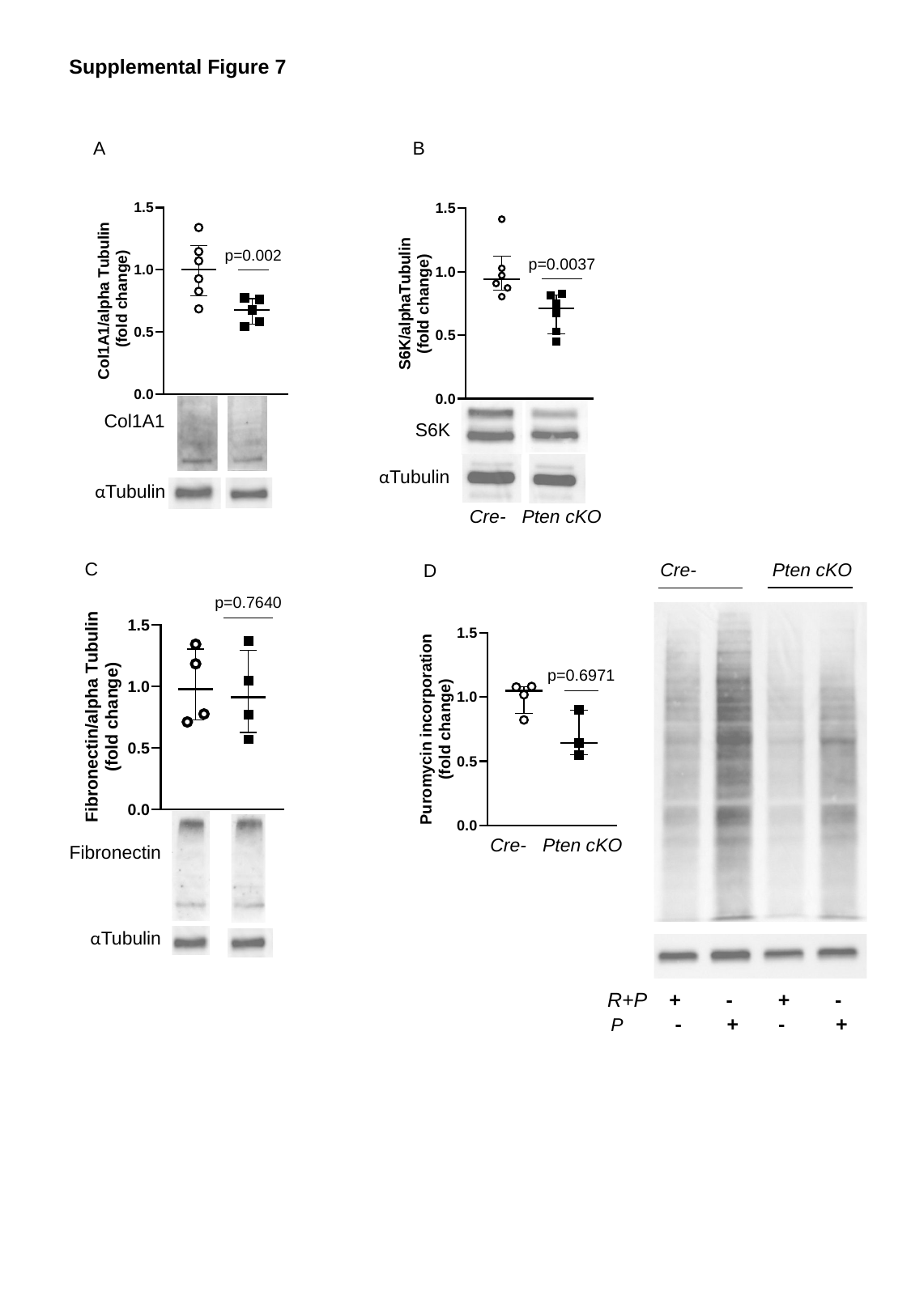

Supplemental Figure 7
A
B
Col1A1
S6K
αTubulin
αTubulin
Cre-
Pten cKO
C
Cre-
Pten cKO
D
Cre-
Pten cKO
Fibronectin
αTubulin
R+P + - + -
P - + - +

## Slide 8
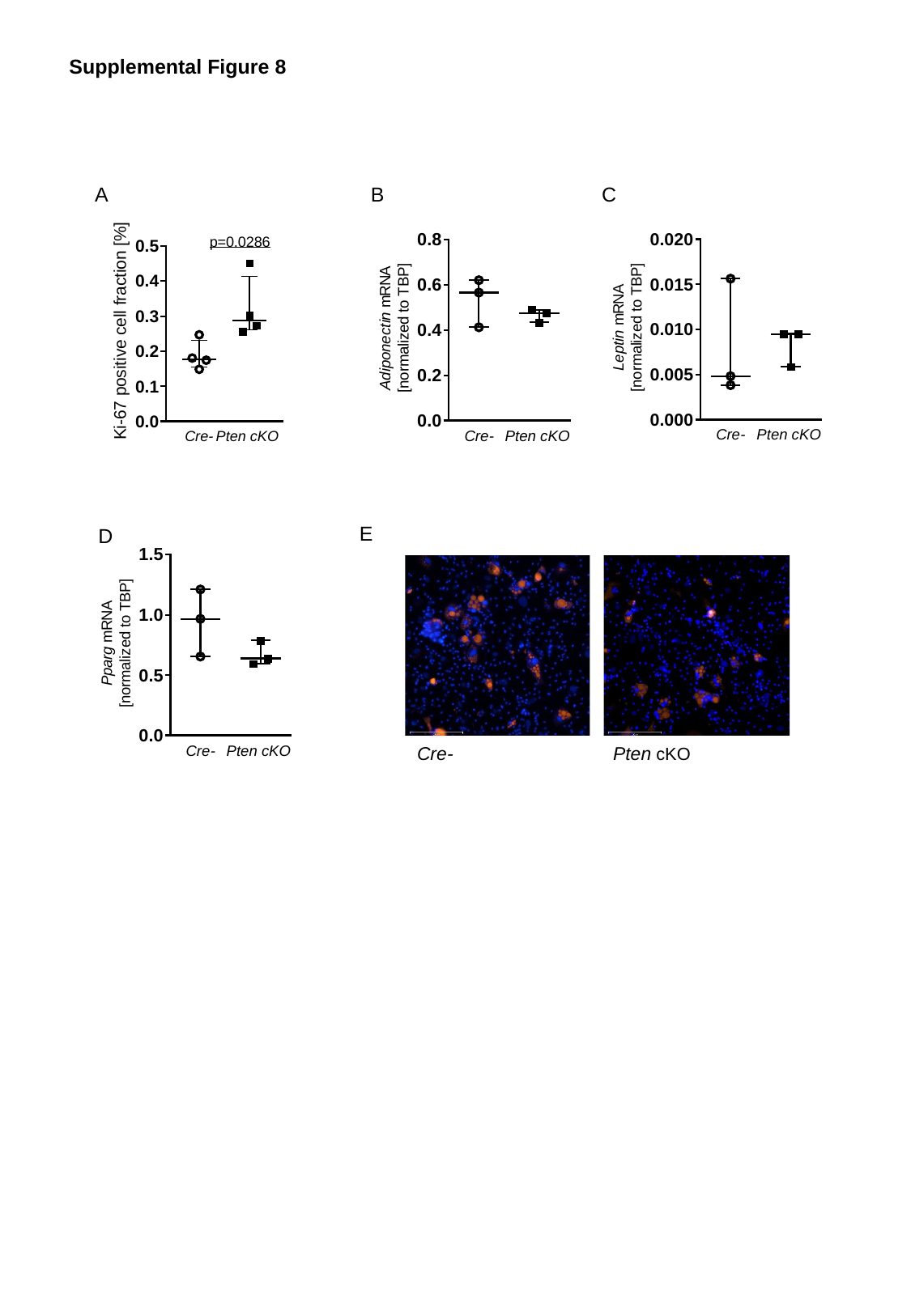

Supplemental Figure 8
A
B
C
E
D
Pten cKO
Cre-
