# Supplementary material for: Pten knockout in mouse preosteoblasts leads to changes in bone turnover and strength
Source: JBMR Plus. 2024 Jan 4;8(3):ziad016. doi: 10.1093/jbmrpl/ziad016 (PMC10945711; doi:10.1093/jbmrpl/ziad016)
Supplement: 231129_Supplemental_Figure_Legends_ziad016 [file 231129_supplemental_figure_legends_ziad016.docx]

**Supplemental Figure 1: Pten knockout enhanced PI3K signaling in bone lysates and BMSCs**

A**)** *Pten* exon 5 floxed or cut-out in gDNA samples from Cre-negative and Pten cKO mouse bone marrow stromal cells (BMSCs). BMSCs from Cre-negative mice show a band at 1100 bp indicating the flowd exon 5 of Pten, while Cre-positive BMSCs show additional band at 400 bp indicating the lack of exon 5. Pten exon 5 knockout was shown in Pten cKO, but not in Cre negative BMSCs.

B, C) Western blot analysis of male Cre-negative (n = 6) and Pten cKO (n = 2) and female Cre-negative (n = 2) and Pten cKO (n = 4) whole bone lysates (whole data): Pten was reduced (normalized to αTubulin), AKT phosphorylation (S473) was elevated (normalized to total AKT).

Western blot analysis of D) male and E) female Cre-negative (n = 3) and Pten cKO (n = 3) BMSCs (whole data): Pten was reduced (normalized to αTubulin), AKT phosphorylation (S473) was elevated (normalized to total AKT).

**Supplemental Figure 2: Description of Pten cKO mouse model**

A) Pten cKO mice showed enlarged spleens in 17 week old male mice (n=4 Cre-negatives, 5 Pten cKO; p=0.111), 17 week old female mice (n=4, p=0.0159) and 38 week old male mice (n=4, p=0.0286).

B) Pten cKO mice have a higher number of lymphocytes and (F) lower neutrophils compared to Cre negative mice (n= 2 Cre negatives, 4 Pten cKO).

Data are presented as median and interquartile range.

**Supplemental Figure 3: Gene expression differences in BMSCs from Pten cKO and control mice (female data)**

Gene expression of A) Osx1, B) Runx2, C) Glut1, D) Pgk, E) Collagen1A1 and F) Cre recombinase in control and Pten cKO bone marrow stromal cells (BMSCs) from female mice (n = 6 Cre negative and n = 5-6 Pten cKO BMSCs). Data are presented as median and interquartile range.

**Supplemental Figure 4: *Pten* cKO in osteoprogenitor cells results in changes of bone architecture of female mice**.

In femoral trabecular bone (n=4 Cre negatives and n=3 Pten cKO), bone architecture was assessed using µCT. Data are presented as median and interquartile range showing all data points.

A) Bone volume/total volume (BV/TV) (p=0.0571) and (B) bone mineral density (BMD) (p= 0.1143) in trabecular bone were increased in Pten cKO bones. C) Femoral trabecular thickness (Tb.Th. Trab.), D) trabecular separation (Tb. Sp. Trab) and E) trabecular number (Tb.N. Trab.) in *Pten* cKO bones was not different compared to controls. While F) cortical BV/TV and G) cortical thickness (Ct. Th. Cort.) were not changed, H) cortical BMD tended to be lower (p=0.0571) in *Pten* cKO bones. Data are presented as median and interquartile range.

**Supplemental Figure 5: *Pten* cKO increases bone turnover and influences bone remodeling (female data).**

Serum markers for bone turnover were quantified using ELISA. (A) Serum Procollagen type 1 N propeptide (P1NP) as a marker for bone formation was elevated (p=0.0095, n=6 Cre negatives, n=4 *Pten* cKO). (B) C-terminal telopeptide of type 1 collagen (CTX) as a marker for bone resorption was increased (p=0.019, n=6 Cre negatives, n=4 *Pten* cKO). (C) Number of osteoblasts/bone perimeter (N.Ob/B.Pm) and (D) number of osteoclasts/bone perimeter (N.Oc/B.Pm) were not significantly different. Data are presented as median and interquartile range.

**Supplemental Figure 6: *Pten* cKO bone phenotype and mechanical properties of bones from female mice**

A) Pten cKO bones are paler than Cre-negative (Cre-) bones. A representative image of 17-week old mice is shown. B) Integrated Density was assessed using Image J and is higher in Pten cKO bones (p=0.0286).

C-E) Mechanical properties of the femoral shaft were assessed by 3-point-bending (n= 4 Cre-negatives, n=3 Pten cKO females per group). Improved mechanical strength was found in *Pten* cKO femora with C) increased elastic modulus (Emod) (p=0.0571), D) higher load or maximum force (Fmax, p=0.1143), and E) significantly increased work to fracture (W to Fmax, p=0.1143).

Quantification of F) osteoblast (Ob.S/BS) and G) osteoclast (Oc.S/BS) number of cortical bone by TRAP staining revealed no significant differences.

Analysis of non-mineralized bone/osteoid in the cortical bone by von Kossa/van Gieson staining. No differences in H) cortical osteoid width, I) osteoid volume (cort. OV/TV) and J) osteoid surface (cort. OS/BS) were observed.

Data are presented as median and interquartile range.

**Supplemental Figure 7: Matrix protein expression and protein synthesis are lower in Pten cKO BMSCs.**

A) Collagen1A1 (Col1A1) protein and B) ribosomal S& kinase (S6K) protein was less expressed in Pten cKO BMSCs than in Cre negative (Cre-) controls (p=0.002 for Col1A1, p=0.0037 for S6K, n=6 Cre-, n=5-6 Pten cKO). C) There was no difference in Fibronectin expression. D) Puromycin incorporation into nascent polypeptides after mTOR inhibition was slightly lower in Pten cKO BMSC lysates (p=0.6971, n=4 Cre-, n=3 Pten cKO), R+P: incubation with 100 nM rapamycin and 1 µM Puromycin, R: incubation with 1 µM Puromycin. Data are presented as median and interquartile range fold changes after normalization to the mean of Cre- values. One representative Western blot image is shown. αTubulin was used as loading control.

**Supplemental Figure 8: Proliferation of BMSCs from female mice and adipogenesis**

(A) Proliferation marker Ki-67 immunofluorescence staining (Ki67/Hoechst: proliferative fraction in %) in Cre negative (Cre-) and Pten cKO BMSCs from female mice at day 1 of proliferation: Pten cKO cells show a higher fraction of Ki-67 positive cells (n=4, p=0.0286).

Expression of adipogenic markers B) Adiponectin (n=3), C) Leptin (n=3) and D) Pparg (n=3), determined using qPCR, did not show any differences at mRNA level in Pten cKO BMSCs. Data are presented as median and interquartile range.

E) Two representative images from control and Pten cKO BMSCs after adipogenic differentiation and stained with Nile Red (lipids) and Hoechst (cell nuclei). Scale bar = 200 µm
